# Analysis of high-risk human papillomavirus infections and cervical intraepithelial neoplasia: factors influencing awareness among women of childbearing age in southwest China

**DOI:** 10.1017/S1463423624000331

**Published:** 2024-10-07

**Authors:** Kexue Ning, Jing Gong, Xianghua Li, Lijuan He

**Affiliations:** 1 College of Agroforestry and Health, The Open University of Sichuan, Chengdu, China; 2 Rural Education and Industrial Revitalization Research Center, The Open University of Sichuan, Chengdu, China; 3 Health Management Center, The Affiliated Hospital, Southwest Medical University, Sichuan, China

**Keywords:** awareness, cervical diseases, high-risk HPV, influential factors, southwest China

## Abstract

**Background:**

High-risk Human Papillomavirus (HPV) infections are a leading cause of cervical diseases among Han Chinese women of reproductive age. Despite studies like Mai *et al*. (2021) addressing HPV prevalence in Southern China, awareness remains low, especially in Southwest China. Our study addresses this gap.

**Objective:**

This hospital-based, retrospective study analyzes the prevalence of high-risk HPV and its association with cervical intraepithelial neoplasia (CIN) among Han Chinese women of reproductive age in Southwest China.

**Methods:**

Data were collected from 724 women undergoing routine health exams from December 2022 to April 2023. A total of 102 women with high-risk HPV infections were identified. A survey assessed HPV awareness, CIN incidence, and socio-demographic factors influencing awareness.

**Results:**

Of the 724 women, 102 (14.1%) were diagnosed with high-risk HPV, with HPV-16 being the most common subtype (22.5%). Awareness was significantly lower among unmarried women (OR: 6.632, *p* = 0.047), those with high school education or less (OR: 20.571, *p* = 0.003), and rural residents (OR: 19.483, *p* = 0.020). HPV-16 was detected in 54.55% of women with high-grade CIN.

**Conclusion:**

There is an urgent need for targeted education and HPV vaccination in Southwest China, particularly for women with lower education, rural residents, and older individuals. Subtype-specific strategies are essential for preventing and managing CIN.

## Introduction

Cervical cancer, primarily caused by persistent infections with high-risk human papillomavirus (hrHPV), continues to be a major global health concern. Human papillomavirus (HPV) types 16 and 18 are recognized as the leading contributors to cervical cancer, accounting for nearly 70% of cases globally (Walboomers *et al*., 1999; Burd, 2003; Bouvard *et al*., 2009; Rodríguez *et al*., 2010). In China, cervical cancer is the second most common cancer among women aged 15–44 years (Chen *et al*., 2016).

While comprehensive data specific to the prevalence of HPV in southwest China are lacking, studies from other regions, such as Shenzhen in Southern China, indicate significant HPV prevalence rates among women with cervical lesions. For example, Mai *et al*. (2021) reported a prevalence rate of 17.83% in Shenzhen, highlighting the importance of addressing HPV infections in urban Chinese populations.

Awareness of hrHPV and its implications for cervical cancer development is crucial for effective prevention strategies. However, there is a notable gap in awareness and understanding among Chinese women, particularly regarding the benefits of HPV vaccination and regular cervical screenings (Schiller and Lowy, 2012; Zhang *et al*., 2016; Chen *et al*., 2018; Zhang *et al*., 2022).

This study aims to investigate socio-demographic and behavioural factors influencing awareness and knowledge of hrHPV infection and cervical diseases among women of reproductive age in China, focusing on developing targeted public health interventions. By analysing prevalent HPV subtypes and their impact on cervical health, this study will provide essential insights to enhance HPV-related health education and vaccination strategies, particularly in regions with limited data like southwest China.

## Materials and methods

### Study design and data collection

This retrospective study was conducted at the health examination centre of a tertiary hospital in southwest China, focusing on Han Chinese women of childbearing age. The aim was to perform an in-depth analysis of the prevalent hrHPV subtypes and the stages of cervical epithelial conditions diagnosed in these women. Data collection occurred between December 2022 and April 2023, sourcing records from the hospital’s database. These records included demographic information such as age, marital status, education level, and place of residence, along with clinical data comprising economic status and the specifically diagnosed hrHPV subtypes. The study was approved by the hospital’s Ethics Committee, ensuring adherence to ethical standards in the handling and analysis of patient data.

### HPV detection and typing

All patients in the cohort underwent hrHPV testing and typing as part of their routine diagnostic process. These procedures were performed using the polymerase chain reaction technique on cervical swab specimens, enabling the identification of specific hrHPV subtypes.

### Cervical intraepithelial neoplasia classification

Patients were categorized based on their diagnosed conditions of cervical intraepithelial neoplasia (CIN) as recorded in their medical files. The classifications followed include: No Intraepithelial Neoplasia or Malignant Lesion (negative for intraepithelial lesion or malignancy); Atypical Squamous Cells of Undetermined Significance (ASC-US); Low-grade Squamous Intraepithelial Lesion (LSIL) encompassing Cervical Intraepithelial Neoplasia 1 (CIN 1); and High-grade Squamous Intraepithelial Lesion (HSIL), including Cervical Intraepithelial Neoplasia 2 and 3 (CIN 2, CIN 3). This approach ensures a focused and accurate assessment of the severity of cervical epithelial abnormalities.

### Assessment of knowledge on HPV and CIN

Knowledge and awareness of HPV infection and CIN were evaluated using the ‘Questionnaire on the Status and Awareness of Cervical High-risk Human Papillomavirus (HPV) Infections and Cervical Intraepithelial Neoplasia among Women of Childbearing Age’. This instrument, administered during hospital visits, was designed to probe participants’ understanding of HPV and its link to CIN, assessing risk factors, prevention, and treatment strategies. The questionnaire featured a series of multiple-choice questions and scales to measure the depth of knowledge comprehensively. Responses were analysed and scored, classifying participants into ‘High Awareness’ and ‘Low Awareness’ groups based on their answers, which facilitated a focused and binary statistical analysis of knowledge levels among the study population.

### Statistical analysis

We utilized descriptive statistics to summarize the demographic and clinical characteristics of the participants. Chi-square tests were used to explore the relationships between different characteristics and HPV and cervical disease knowledge. Univariate and multivariate logistic regression models were used to identify the factors associated with higher knowledge levels. A *p*-value < 0.05 was considered statistically significant. All statistical analyses were carried out using R software.

## Results

### Demographic and socioeconomic characteristics of the study participants

Table 1 summarizes the demographic and socioeconomic profiles of 102 women of reproductive age who are at high risk for cervical HPV infection. The data delineate a diverse group predominantly comprised of unmarried women, reflecting varied educational backgrounds predominantly at or below high school level. The employment status of participants spans several categories, with a notable number engaged in casual work, administrative roles, or self-employment. Residency splits nearly evenly between urban and rural settings, indicating a broad geographical representation within the study cohort. The analysis also highlights significant variations in household income and demonstrates a general trend of low awareness about high-risk cervical HPV infection among the participants. Further details and specific percentages are available in Table 1.

**Table 1.** Demographic and socioeconomic characteristics of the study participants

Characteristics	Overall
Age, median (IQR)	31.5 (27, 35)
Marital status, n (%)	
Married	26 (25.5%)
Unmarried	76 (74.5%)
Education level, n (%)	
College and below	22 (21.6%)
High school	32 (31.4%)
Junior high and below	48 (47.1%)
Occupation, n (%)	
Administration	22 (21.6%)
Company	16 (15.7%)
Casual worker	28 (27.5%)
Others	14 (13.7%)
Individual	22 (21.6%)
Residence, n (%)	
Urban	50 (49%)
Rural	52 (51%)
Household income, median (IQR)	2953 (2219.5, 4362)
Science knowledge, n (%)	
High	29 (28.4%)
Low	73 (71.6%)

*Note*: Table presents the overall characteristics of the study participants. Age and Household Income are presented as medians with interquartile range (IQR). Marital Status, Education Level, Occupation, Residence, and Science Knowledge are presented as numbers with percentages. Percentages might not add up to 100% due to rounding.

### Comparison of characteristics between high- and low-awareness groups for high-risk cervical HPV infection

Table 2 outlines the disparities between women with high- and low-awareness of high-risk cervical HPV infection. It highlights significant differences in demographic and socioeconomic attributes across the two groups. The analysis shows that younger women tend to have higher awareness levels, with significant differences also noted in marital status, education levels, occupation, place of residence, and household income. Specifically, women in the high-awareness group generally hold higher educational qualifications, are more likely to be married, and reside predominantly in urban areas. Moreover, they demonstrate markedly higher household income and greater knowledge of science related to HPV compared to their counterparts in the low-awareness group. These variations are statistically significant, suggesting strong correlations between these factors and awareness levels of hrHPV infections. Detailed statistical values and comparisons are provided in Table 2.

**Table 2.** Comparison of characteristics between high- and low-awareness groups for high-risk cervical HPV infection

Characteristics	High	Low	*p* Value
n	15	87	
Age, median (IQR)	21 (19.5, 23)	33 (29, 36)	<0.001
Marital status, n (%)			<0.001
Married	10 (9.8%)	16 (15.7%)	
Unmarried	5 (4.9%)	71 (69.6%)	
Education level, n (%)			<0.001
College and below	13 (12.7%)	9 (8.8%)	
High school	2 (2%)	30 (29.4%)	
Junior high and below	0 (0%)	48 (47.1%)	
Occupation, n (%)			<0.001
Administration	10 (9.8%)	12 (11.8%)	
Company	5 (4.9%)	11 (10.8%)	
Casual worker	0 (0%)	28 (27.5%)	
Others	0 (0%)	14 (13.7%)	
Individual	0 (0%)	22 (21.6%)	
Residence, n (%)			<0.001
Urban	14 (13.7%)	36 (35.3%)	
Rural	1 (1%)	51 (50%)	
Household income, median (IQR)	8351 (6761, 9086.5)	2726 (2102, 3697.5)	<0.001
Science knowledge, n (%)			<0.001
High	15 (14.7%)	14 (13.7%)	
Low	0 (0%)	73 (71.6%)	

*Note*: The table compares the characteristics of the study participants between high awareness (n = 15) and low awareness (n = 87) groups for high-risk cervical HPV infection. Age and Household Income are presented as medians with interquartile range (IQR). Marital Status, Education Level, Occupation, Residence, and Science Knowledge are presented as numbers with percentages. Statistical analyses were conducted using Chi-square tests for categorical variables and Mann–Whitney *U*-tests for continuous variables to compare between the high- and low-awareness groups. *p* Values indicate the level of statistical significance for differences between the groups, with a value less than 0.05 considered significant. Percentages might not add up to 100% due to rounding.

### Univariate and multivariate analysis of factors associated with awareness of hrHPV infection and CIN

Table 3 outlines the statistical analyses used to identify factors influencing awareness of hrHPV infection and CIN. The analyses delineate how marital status, education level, and residence significantly affect awareness levels. Notably, being unmarried, having a lower education level, and residing in rural areas are associated with lower awareness. These factors remain significant predictors in both univariate and multivariate models, highlighting their robust impact on awareness disparities among the study population. Specific odds ratios (OR) and statistical significance details are detailed in Table 3, demonstrating the strength and consistency of these associations across different analytical approaches.

**Table 3.** Univariate and multivariate analysis of factors associated with awareness of hrHPV infection and cervical intraepithelial neoplasia

Characteristics	Total (N)	Univariate analysis		Multivariate analysis	
		Odds ratio (95% CI)	*p* Value	Odds ratio (95% CI)	*p* Value
Marital status	102				
Married	26	Reference		Reference	
Unmarried	76	8.875 (2.666–29.547)	**<0.001**	6.632 (1.027–42.807)	**0.047**
Education level	102				
College and below	22	Reference		Reference	
High school	32	21.667 (4.101–114.484)	**<0.001**	20.571 (2.852–148.395)	**0.003**
Junior high and below	48	1234328951.9288 (0.000–Inf)	0.993	812115240.2837 (0.000–Inf)	0.993
Residence	102				
Urban	50	Reference		Reference	
Rural	52	19.833 (2.495–157.645)	**0.005**	19.483 (1.583–239.851)	**0.020**

*Note*: N denotes the total sample size. In the univariate and multivariate analyses, ORs are provided with their respective 95% CIs. The reference category for each variable is indicated in the table. For marital status, ‘Married’ serves as the reference category; for education level, ‘College and Below’ is the reference; and for residence, ‘Urban’ is used as the reference. These categories were used as the baseline for the calculation of ORs in both univariate and multivariate logistic regression analyses. Statistical significance was assessed using logistic regression models, which allow for the adjustment of various covariates in the multivariate analysis. *p* Values < 0.05 are considered statistically significant. ‘Inf’ represents infinity, indicating an undetermined upper limit for the confidence interval in this data due to extreme OR values observed particularly for some categories of educational level.

Bold values indicate statistically significant results (*p* < 0.05).

### Distribution of hrHPV infection subtypes across various categories of cervical epithelial conditions

Table 4 examines the distribution of hrHPV subtypes within different cervical epithelial conditions, categorized as No Intraepithelial Neoplasia or Malignant Lesion, Undetermined Significance of Atypical Squamous Epithelium, LSIL, and HSIL. The analysis reveals the varying prevalence of each subtype across these categories, highlighting specific patterns of distribution. For example, HPV-16 and HPV-52 emerge as significant subtypes, showing varying prevalences that suggest differing pathological impacts on cervical epithelial conditions. Detailed percentages and case numbers for each subtype and category are elaborated in the table, providing a comprehensive overview of the landscape of HPV subtype infection in relation to cervical disease severity.

**Table 4.** Distribution of hrHPV infection subtypes across various categories of cervical epithelial conditions

HPV subtype of infection	Total cases	No intraepithelial neoplasia or malignant lesion	Undetermined significance of atypical squamous epithelium	Low-grade squamous intraepithelial lesion (LSIL)	High-grade squamous intraepithelial lesion (HSIL)
HPV-52	15	8 (21.05)	5 (13.16)	2 (13.33)	0 (0.00)
HPV-16	23	4 (10.53)	7 (18.42)	6 (40.00)	6 (54.55)
HPV-58	12	4 (10.53)	5 (13.16)	3 (20.00)	0 (0.00)
HPV-56	8	4 (10.53)	2 (5.26)	2 (13.33)	0 (0.00)
HPV-39	8	3 (7.89)	5 (13.16)	0 (0.00)	0 (0.00)
HPV-51	7	3 (7.89)	3 (7.89)	1 (6.67)	0 (0.00)
HPV-33	7	3 (7.89)	2 (5.26)	0 (0.00)	2 (18.18)
HPV-31	6	3 (7.89)	1 (2.63)	0 (0.00)	2 (18.18)
HPV-18	6	2 (5.26)	3 (7.89)	1 (6.67)	0 (0.00)
HPV-68	4	1 (2.63)	2 (5.26)	0 (0.00)	1 (9.09)
HPV-59	3	2 (5.26)	1 (2.63)	0 (0.00)	0 (0.00)
HPV-35	2	1 (2.63)	1 (2.63)	0 (0.00)	0 (0.00)
HPV-45	1	0 (0.00)	1 (2.63)	0 (0.00)	0 (0.00)

*Note*: This table represents the distribution of different hrHPV subtypes across various categories of cervical epithelial conditions. Number of cases and percentage (%) are indicated. Percentages are calculated with respect to the total number of cases within each category. The categories are No Intraepithelial Neoplasia or Malignant Lesion, Undetermined Significance of Atypical Squamous Epithelium, LSIL, and HSIL. The number of cases for each HPV subtype and the percentage distribution in each category are provided. Please note that the percentages are calculated as a proportion of the total number of cases for each HPV subtype.

## Discussion

Our study results underscore a significant lack of awareness regarding high-risk HPV infection and cervical diseases among women of reproductive age in southwest China, echoing prior studies that report a general HPV knowledge deficiency (Zhang *et al*., 2016; George *et al*., 2020; Chido-Amajuoyi *et al*., 2021). Notably, age, marital status, education level, urban or rural residence, income, and scientific literacy emerged as significant factors influencing these awareness levels. Furthermore, the data revealed unique HPV subtype prevalence across various cervical conditions, providing valuable insights for potential preventive strategies and interventions.

Our analysis revealed a stark association between socio-demographic attributes, such as marital status and education level, and the awareness level of the participants. Both unmarried women and those who had attained a high school education or less were found to have a significantly higher likelihood of displaying low awareness, compared to their respective counterparts. This observation resonates with previous research that has similarly identified marital status and educational level as significant predictors of HPV awareness and vaccine acceptability (Adegboyega *et al*., 2023; Hailu *et al*., 2023).

Importantly, our findings indicate age as a key determinant of HPV awareness. Younger women demonstrated higher levels of knowledge regarding HPV and associated cervical diseases. This could be attributed to the advent of newer, more comprehensive sexual health education programmes that are more accessible to the younger generation (Chesser *et al*., 2016). Moreover, younger women tend to be more familiar with digital technologies, including the internet and social media platforms, which have been increasingly employed in recent years for health education and promotion (Gough *et al*., 2017). In contrast, older women presented a more worrying gap in awareness. This could be attributed to a lack of targeted educational initiatives for this age group, who may also have had limited exposure to sexual health education during their school years. Furthermore, older women may be less comfortable or adept at accessing digital health resources. There is also the possible effect of age-related cognitive decline, which can affect the retention and understanding of new health-related information (Tuohy *et al*., 2019). This highlights a pressing need for the development of tailored, age-specific HPV education interventions. Particularly for older women, these interventions could involve more traditional and familiar modes of delivery, such as print media and face-to-face health talks, and should ideally be embedded within routine health services to ensure optimal reach and impact (Kruk *et al*., 2018).

Additionally, the location of residence was another significant determinant of HPV awareness, with women residing in rural areas exhibiting an alarmingly higher risk of low awareness compared to their urban counterparts. This finding is congruent with prior research highlighting the critical disparity in HPV awareness and cervical cancer screening between rural and urban women, attributable to the unavailability or inaccessibility of health services and educational resources in rural areas (Johnson *et al*., 2018; Rosser *et al*., 2015).

Interestingly, our study found HPV-16 to be the most prevalent subtype, especially among the cases with high-grade intraepithelial neoplasia. This finding is consistent with the global trend of HPV-16 being the leading cause of cervical cancer worldwide (Choi *et al*., 2023). The identification of HPV-16 as the leading subtype underlines the necessity of a vaccination programme targeting this subtype for the efficient prevention of cervical cancer.

While our study provides important insights, it is not without limitations which must be acknowledged when interpreting our findings. Firstly, the assessment of participants’ knowledge about HPV and cervical diseases was estimated based on documented interactions and health education provided during their hospital visit. However, this estimation might not fully capture the complexity of each patient’s understanding, and their individual interpretation could add complexity to our findings (Althubaiti, 2016).

Secondly, the generalizability of our findings may be limited as our sample was solely drawn from the southwest region of China. This geographic specificity might influence the results. The levels of knowledge about HPV and cervical diseases observed in our sample might have been influenced by the specific socio-cultural environment and available healthcare resources in this region. These factors might not be representative of other regions in China, which could have different cultural attitudes towards cervical cancer and access to different healthcare resources.

Thirdly, despite our sample size being adequate, it might not have been large enough to capture the broad spectrum of socio-demographic factors influencing awareness about HPV infection and cervical diseases. A larger and more diverse sample might have provided more robust and generalizable results.

Lastly, while this study has identified certain socio-demographic factors associated with awareness, it is likely that there are other unidentified factors, such as psychological, cultural, or environmental elements, which might influence an individual’s knowledge and perceptions about HPV infection and cervical diseases. Future research might benefit from considering these aspects to provide a more comprehensive understanding of the determinants of HPV awareness. These limitations underscore the necessity of further research, incorporating a broader geographical scope and diverse methodologies, to corroborate and extend our findings.

## Conclusions

Our findings provide crucial insights for the development of targeted interventions aimed at improving awareness about HPV infections and CIN in the southwest region of China. The results underscore the need for public health strategies that account for the socio-demographic characteristics of the target population, as well as the necessity of expanding vaccination programmes to cover prevalent HPV subtypes.

## Data Availability

The datasets analysed during the current study are not publicly available due to privacy but are available from the corresponding author at a reasonable request.
